# Evaluating Classifiers to Detect Arm Movement Intention from EEG Signals

**DOI:** 10.3390/s141018172

**Published:** 2014-09-29

**Authors:** Daniel Planelles, Enrique Hortal, Álvaro Costa, Andrés Úbeda, Eduardo Iáñez, José M. Azorín

**Affiliations:** Brain-Machine Interface Systems Lab, Miguel Hernández University of Elche, Avda. de la Universidad S/N, 03202, Elche (Alicante), Spain; E-Mails: acosta@umh.es (A.C.); aubeda@umh.es (A.U.); eianez@umh.es (E.I.); jm.azorin@umh.es (J.M.A.)

**Keywords:** brain-computer interface, ERD, movement intention, classifier

## Abstract

This paper presents a methodology to detect the intention to make a reaching movement with the arm in healthy subjects before the movement actually starts. This is done by measuring brain activity through electroencephalographic (EEG) signals that are registered by electrodes placed over the scalp. The preparation and performance of an arm movement generate a phenomenon called event-related desynchronization (ERD) in the mu and beta frequency bands. A novel methodology to characterize this cognitive process based on three sums of power spectral frequencies involved in ERD is presented. The main objective of this paper is to set the benchmark for classifiers and to choose the most convenient. The best results are obtained using an SVM classifier with around 72% accuracy. This classifier will be used in further research to generate the control commands to move a robotic exoskeleton that helps people suffering from motor disabilities to perform the movement. The final aim is that this brain-controlled robotic exoskeleton improves the current rehabilitation processes of disabled people.

## 1. Introduction

Over the last few years, diverse research has been undertaken in neuroscience in order to obtain a deeper knowledge about the human brain. As a consequence, the development of brain-computer interfaces (BCIs) has increased. A BCI is a system that gives a communication channel between the human brain and a computer [1]. Thus, it provides a new way to interact with the environment, which can be useful for many people who have motor disabilities. These systems are able to capture brain signals using invasive or non-invasive methods and then process these signals in order to generate control commands for a specific device. Invasive techniques register the activity of one neuron or small groups of neurons through microelectrodes implanted in the brain, and they have been used to determinate the movement intention in animals [2] or to control a computer cursor [3]. On the other hand, electroencephalographic (EEG) recordings are non-invasive and allow registering brain activity without surgery. Therefore, these techniques are preferable because of ethical concerns and medical risks. Therefore, this paper uses non-invasive BCI based on electroencephalographic (EEG) signals measured through superficial electrodes placed on the scalp.

Many cognitive processes performed by the brain are well located around the cortex. The occipital lobe is responsible for processing visual information, and it can provide relevant information and a good signal-to-noise ratio, whilst the parietal and frontal lobes take part in intention, planning and the decision to make a movement [4]. There are some extensively studied potentials generated in the occipital area as a response to a visual stimulus, like steady state visually evoked potential (SSVEP) or P300 potential, a kind of event related potential (ERP) wave [5]. These potentials can be used to know where the subject is focusing his gaze, and then it can be applied, for example, to control a wheelchair [6,7] or to write words in a web browser [8]. The main problem is that these potentials are evoked, so an external visual stimulation is necessary to provoke them, which implies the necessity of using a graphical interface to interact with, and therefore, this increases the limitations of its application. On the other hand, there is a family of potentials generated on the subject's own will, which are called spontaneous potentials. Current research has already used these kinds of signals to control different devices, e.g., a robotic arm [9,10] or a wheelchair [11].

One of the main objectives of studies based on BCIs is motor rehabilitation. The parietal and frontal lobes are more interesting because the signals acquired from them allow knowing when an arm movement will be performed before it actually starts, due to their relation with motor action. In this case, it is known that when a person is going to perform a movement, the body runs a chain of events that ends with the action of the muscles and, therefore, the actual movement [12]. This chain starts in the brain only a few tenths of a second before the movement onset, and after that, the electrical signal passes through the spinal cord and reaches the muscles that exert the necessary force. Current technology allows collecting and processing electroencephalographic (EEG) signals from the cortex even in a real-time application, and therefore, a wide range of applications can be developed.

On this study, we are interested in detecting an arm movement before it actually happens. There are two appropriate neuro-physiological phenomena that begin before a voluntary action occurs, and they have different sources [13]. On the one hand, there is a slow potential called the Bereitschaftspotential (also known as the readiness potential), which manifests as a decrement in the closest frequencies to the DC component in EEG signals [14]. This occurs in two phases: the first one with a small decrease of voltage, which starts around 1.5 s before the movement onset, and the second one with a pronounced decrease, which starts around 0.5 s before the movement. On the other hand, the event-related desynchronization (ERD) refers to a decrease in the spectral power of EEG signals in the mu and beta frequency bands [15]. This one starts up to two seconds before the movement onset, and it ends approximately when the movement is finished. After that, the spectral power recovers its magnitude, generating the event-related synchronization (ERS). Some studies have already used these phenomena to know the intention of movement, such as to anticipate a wrist movement [16,17] or an ankle movement [18]. In these cases, the movement to perform was not complex, but, for example, in [19], a reaching movement that requires the use of several muscles and coordination is studied. Moreover, there is research related to the lower limb, where the gait is under consideration [20].

The study and detection of electrical signals that occur in the brain just before undertaking a particular movement can be very useful to assist the movement of people with some motor disabilities that make it difficult or impossible for them to do the action on their own. A way to help these people could be through an exoskeleton attached to the impaired segment of their body [21]. The orthosis releases the effort from the patient's muscles. The user will think about starting an arm movement, and a suitable processing and classification of the EEG signals will detect it before happening. The classifier output could be used to activate the exoskeleton engines, in the case of an active exoskeleton [22]. Furthermore, it could activate a functional electrical stimulation (FES) to stimulate specific muscles when the exoskeleton is passive [23]. Therefore, users will perform the movement when they wish, namely when the classifier detects a pre-movement. In a motor rehabilitation process, this coordination between the desire to execute a movement and the performance of the action might improve the effects of rehabilitation. Nowadays, exoskeletons have been used in research to help with different tasks. The Armeo Spring system is an upper limb orthosis, which can be a tool to train people that suffer multiple sclerosis [24]. In the project, WOTAS (wearable orthosis for tremor assessment and suppression) [25], another system is able to suppress tremors in the arm. Moreover, there are exoskeletons for lower limb rehabilitation, like those developed in the HYPER project, hybrid neuroprosthetic and neurorobotic devices for functional compensation and rehabilitation of motor disorders [26].

In this paper, a methodology to detect the intention of doing a reaching movement with the upper limb using a non-invasive system with spontaneous EEG signals based on the ERD phenomenon is presented. The main objective of this paper is to make a comparison between classifiers in order to choose the most convenient to detect the movement intention. The best classifier will be used for further research. The final goal is to obtain a system to activate FES or the exoskeleton engines to move the arm of those people who have motor damage. Moreover, an exoskeleton attached to the upper limb will be used to support the weight of the weak limb. Both the exoskeleton and FES are not discussed in this paper. The current research is developed as a part of the project, Brain2Motion–Development of a Multimodal Brain-Neural Interface to Control an Exoskeletal–Neuroprosthesis Hybrid Robotic System for the Upper Limb (DPI2011-27022-C02-01), funded by the Spanish Ministry of Economy and Competitiveness.

The remainder of the paper is organized as follows. In Section 2, the performed experiment is explained. In Section 3, the results obtained with several classifiers are shown and discussed, and Section 4 contains the conclusions and future work.

## 2. Experimental Section

### 2.1. Register

During the experimental tests, a commercial amplifier (g.USBamp, g.Tec, GmbH, Austria) was used. The amplifier has 16 channels and also an independent reference and ground input. The EEG signals acquired by the amplifier were registered with a 256-Hz sampling frequency. A computer software developed in MATLAB (software Matrix Laboratory of MathWorks) read and processed the data acquired. The MATLAB API (application programming interface) provided with the amplifier was used to manage it.

**Figure 1.**
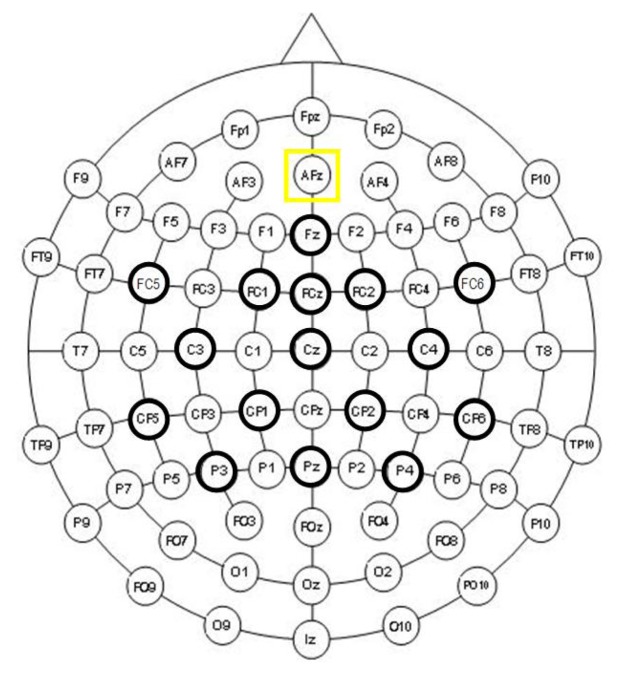
Placement of the electrodes according to the International 10/10 System.

The acquisition of EEG signals was done using 16 active Ag/AgCl electrodes distributed over the scalp. The distribution of sensors on the scalp of the subject was: Fz, FC5, FC1, FCz, FC2, FC6, C3, Cz, C4, CP5, CP1, CP2, CP6, P3, Pz and P4, while the mono-auricular reference was placed on the right ear lobe and the ground was located on AFz, according to the International 10/10 System (Figure 1). To ensure a better placement of the electrodes, a cap (g.GAMMAcap, g.Tec, GmbH, Austria) was used. Furthermore, this system is able to reduce electromagnetic interference.

**Figure 2.**
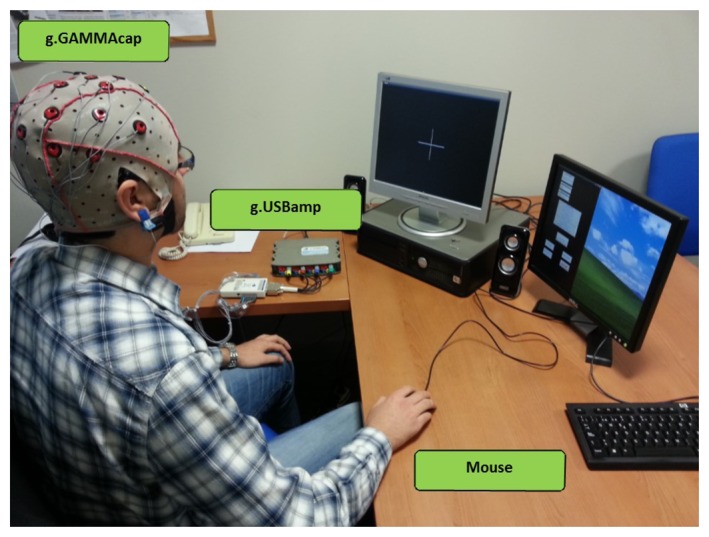
Experimental set up. The subject was wearing a cap with 16 electrodes and he/she had to move the mouse forward/backward. EEG signals were collected and registered by the amplifier.

In the experimental tests, the user had to move a computer mouse. The position of the mouse in the computer screen determines the beginning and also the phase of the movement. A sampling frequency of 16 Hz was used to register the mouse position. The complete set up can be seen in Figure 2.

### 2.2. Test Description

**Figure 3.**
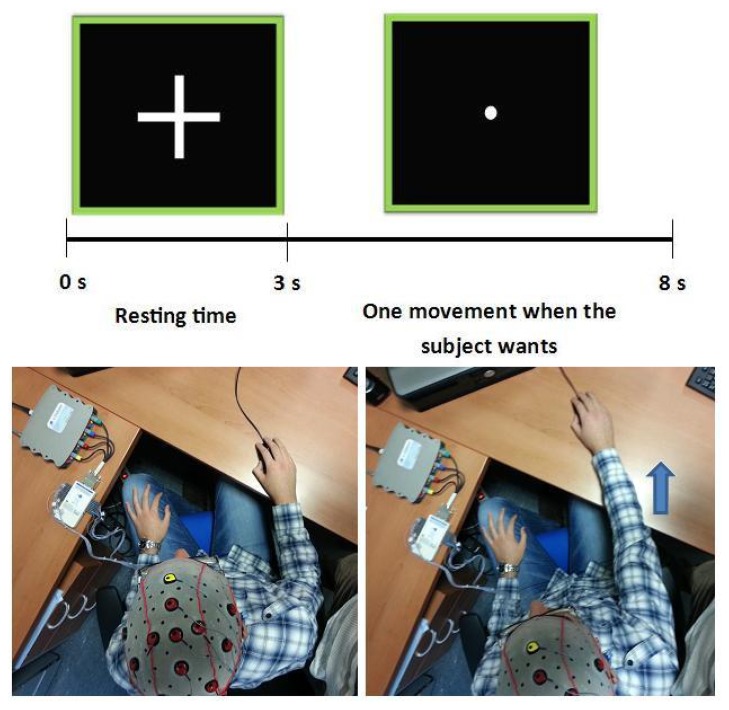
Description of the experiment. Resting position (**bottom left**) and final position of the movement (**bottom right**).

The experiment was performed by 6 healthy subjects between 23 and 31 years old (26.17 ± 3.31 average), all men and right-handed. All volunteers had normal vision and hearing and no history of neurological or psychiatric disorders. The test was done in an isolated room to prevent noise and distractions. Each subject was instructed to perform a reach movement forward and backward with the mouse and then return to the starting position. A graphical interface was used to guide the subject on each performance, and it was used to separate the data between resting and movement time. The interface showed a cross for 3 s when the subject had to remain at rest with the cursor at the bottom of the screen, showing later a point during 5 s. During this period, the subject could freely make a movement and went back to the initial position (Figure 3). Users were warned not to start the movement immediately when the point was shown (at least 1 s later), because EEG signals could be affected by visual stimulus. All subjects have performed one session with 6 runs. Each run consisted of 256 s.

### 2.3. Data Selection

The first step to carry out in the analysis was to select the data. In our study, it was necessary to differentiate between pre-movement data, which was obtained in a 1-s window width before the beginning of the arm movement, and resting data, which was obtained in a 1-s window width when the subject had to keep calm.

From the position of the mouse, it was possible to know the position of the arm. The subject had to remain immobile with the cursor at the bottom of the screen and then move the mouse forward to the top, then backward to the initial position. Therefore, the time instant of the movement initiation was when the mouse changed its position, and the data to analyze were 1 s before it. On the other hand, the resting data were defined as 1 s in the middle of a period when the interface showed a cross.

### 2.4. Signal Pre-Processing

The EEG signals are of the order of microvolts, and due to their poor signal-to-noise ratio, it was necessary to use some filters to improve their quality. Firstly, two frequency filters were applied. One of them was a 50-Hz notch filter to eliminate the power line interference. This filter was designed using an internal hardware filter in the amplifier device. After that, an 8th order Butterworth filter programmed in MATLAB from 5 to 40 Hz was applied to remove some artifacts and the DC component, preserving only the information of the interesting frequencies, which are the mu and beta frequency bands (8–30 Hz).

Secondly, a spatial filter was applied on all EEG channels to reduce the contribution of the remaining electrodes in each channel and, therefore, to isolate better the information collected from each position [27]. A Laplacian algorithm was applied for all electrodes. This algorithm uses the information received from all of the remaining electrodes and their distances from them. The visual result is a smoother time signal, which should contain only the contribution coming from the particular position of the electrode. The Laplacian was computed according to the formula:

(1) $$Vi^{LAP}=Vi^{CR}-\sum_{j\in Si}g_{ij}Vj^{CR}$$

where *Vi^LAP^* is the result of applying this algorithm to the electrode *i* , *Vi^CR^* is the electrode *i* signal before the transformation and,

(2) $$g_{ij}=\frac{\frac{1}{d_{ij}}}{\sum_{j\in Si}\frac{1}{d_{ij}}}$$

where *Si* contains all of the electrodes, except from the electrode *i* and *d_ij_* is the distance between *i* and *j* electrodes.

### 2.5. Features Extraction and Classifiers

**Figure 4.**
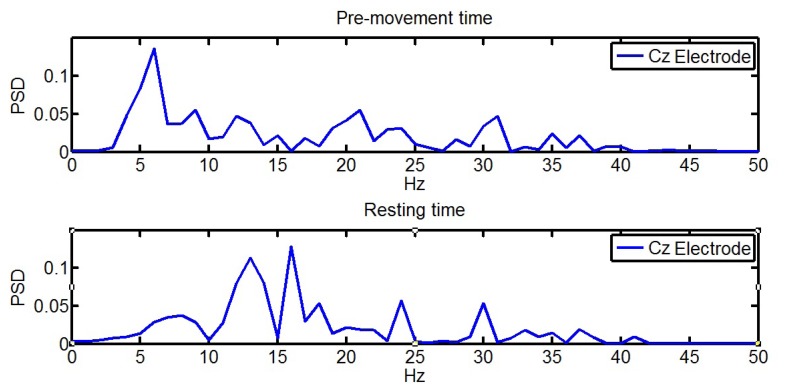
FFT performed on a single pre-movement (**top**) and resting (**bottom**) data of the electrode Cz. In this case, features in pre-movement are 0.1760, 0.2993, 0.0749 and in the sample of resting data are 0.1784, 0.5455 and 0.0722. In pre-movement data, there is lower activity in the 13–24 Hz band sum than in the resting data.

The selected EEG data were processed with a fast Fourier transform (FFT) to know the spectral power. Due to the complexity of detecting the ERD phenomenon in real time based on experiments previously performed, a novel methodology to define the cognitive process was used. This technique allows using data mining and more sophisticated classifiers instead of thresholds. Therefore, the features were the sums of three frequency bands, 8–12 Hz, 13–24 Hz and 25–30 Hz, with a 1-Hz resolution per electrode, which represents the mu and beta bands, making a total of 48 features. Figure 4 shows an FFT output of pre-movement and resting data selected. It is possible to see that, in pre-movement, the power spectra between 11 and 18 Hz is lower than in resting time. These features were inputs to some classifiers typically used in BCI [28,29]. This study analyzes support vector machine (SVM), k-nearest neighbor (k-NN) and naive Bayes (NB) classifiers implemented in MATLAB.

SVM is an approach where the objective is to find the best separation hyperplane, which provides the highest margin distance between the nearest points of two classes to separate them. Typically, a convex quadratic programming (QP) [30] is solved to determine the SVM model, but in this paper, a least squares (LS) SVM [31] method was also used. Moreover, an alternative algorithm to solve the optimization problem in SVM, called sequential minimal optimization (SMO), has been applied [32]. Both LS-SVM, as well as SMO reduce the computational complexity, since the first one transforms the convex quadratic programming (QP) problem into linear equations and the second one subdivides the mathematical problem of QP-SVM into subproblems. Support vector machine classifiers are widely used in BCI systems, and they usually achieve great results [33].

The k-NN classification rule is based on the density estimation using the distance from nearest neighbors [34]. This classifier is non-linear, and its computational complexity is dependent of the number of neighbors. Firstly, a training phase from a given population is done with k as the number of nearest neighbors (in our case, k = 10, 20 and 30) used in the classification. The distance metric can be changed between several methods, such as Euclidean or Hamming, among others.

The classification paradigm that uses Bayes's theorem in conjunction with the conditional independence hypothesis of the predictor variables given the class is known as naive Bayes [35]. This method computes the probability that a sample belongs to a class and assigns it to the most likely class by comparing a linear combination of the features with a threshold.

## 3. Results and Discussion

**Figure 5.**
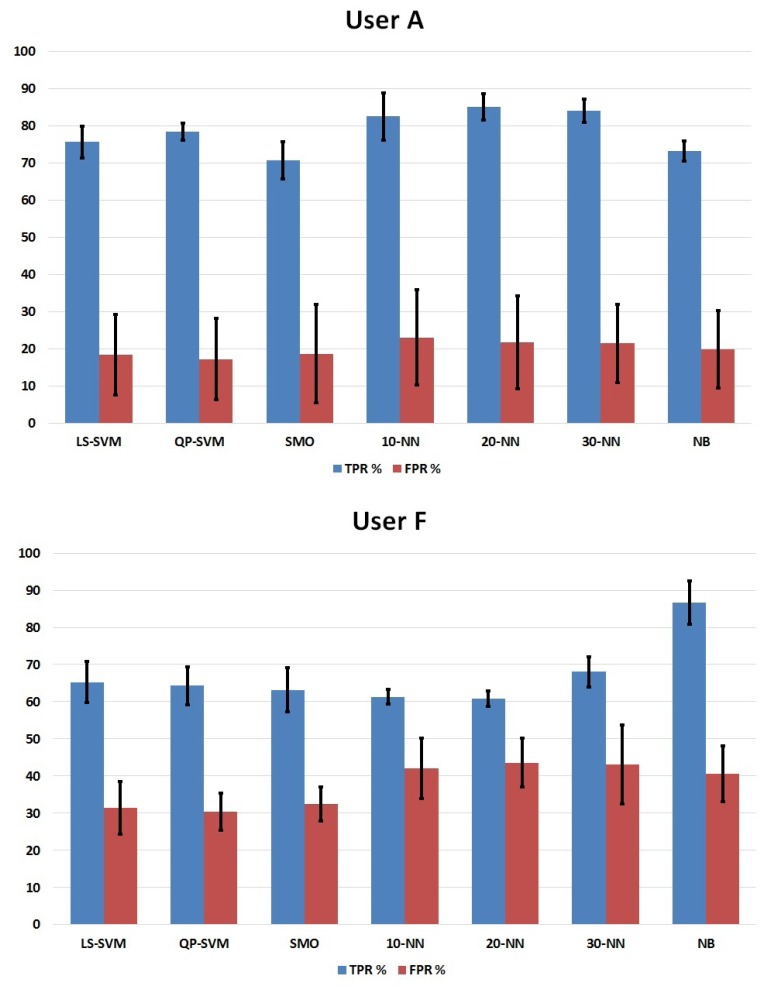
True positive rate (TPR) and false positive rate (FPR) of User A (**top**) and User F (**bottom**) for each classifier.

In this section, seven classifiers were evaluated in order to choose the best one to detect the intention of an arm movement. The classifiers used were LS-SVM, QP-SVM, SMO and k-NN, with 10, 20 and 30 neighbors, and Naive Bayes. The discussion is going to be performed using three parameters: true positive rate (TPR), false positive rate (FPR) and the GAP (TPR divided by FPR) obtained in a six-fold cross-validation. Each run performed was used as a fold; all combinations of five folds were utilized to create a classifier, and the remaining fold was used to test it. The parameters of each user calculated with all iterations of the cross-validation and an average of all users are shown in Table 1. In Figure 5, a bar plot with the results of the best user (A) and the worst user (F) in terms of GAP is shown, too. The values of TPR and FPR were calculated as follows:

(3) $$\text{TPR}=\frac{\text{pre}-\text{movements detected as pre}-\text{movement}}{\text{sum of pre}-\text{movements in test}}*100$$

(4) $$\text{FPR}=\frac{\text{resting samples detecate as pre}-\text{movement}}{\text{sum of resting samples in test}}*100$$

**Figure 6.**
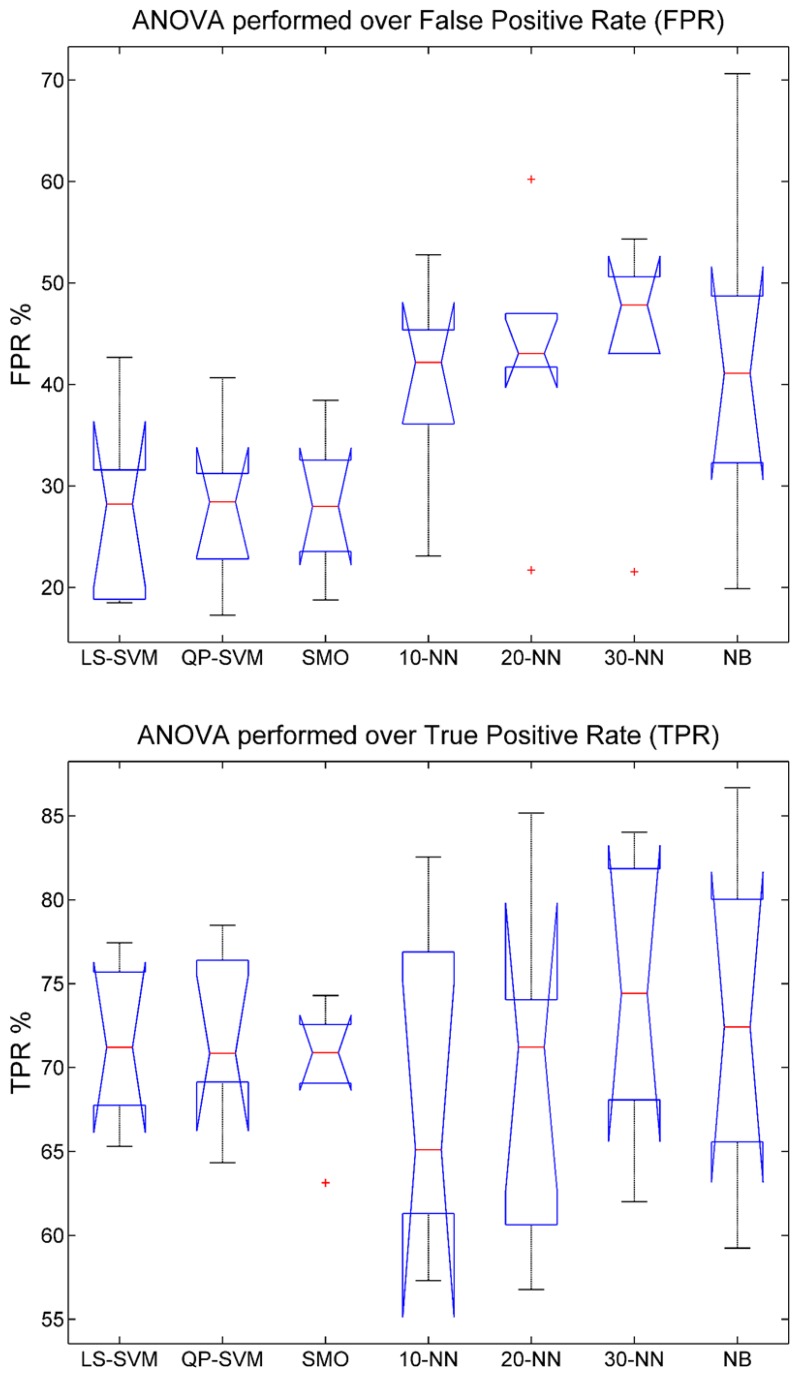
ANOVA results for false positive rate (FPR) (**top**) and true positive rate (TPR) (**bottom**) over all users. *p*-value = 0.0225 and *p*-value = 0.8774, respectively.

In order to analyze the significant differences between classifiers, a statistical study using ANOVA for TPR and FPR has been done (Figure 6). According to the results obtained using TPR, there is no significant difference between classifiers (*p*-value = 0.8774 > 0.05). The interquartile range is the more noticeable characteristic, because SVM classifiers have less range than others. Hence, the SVM family was more stable between users. Regarding FPR, there is a significant difference between classifiers (*p*-value = 0.0225 < 0.05). In order to know the classifiers that obtain a significantly lower FPR index, an ANOVA with all combinations of two classifiers have been performed. The p-value of every combination is shown in Table 2. All SVM classifiers achieve significant differences compared to k-NN (*p*-value < 0.05) and somewhat less significant ones compared to NB (*p*-value < 0.1). The remaining combinations do not show significant differences. Therefore, it is not possible to choose a concrete SVM classifier, but at least this family was better than others. Then, an analysis of the TPR, FPR and GAP indexes obtained was done.

### 3.1. True Positive Rate (TPR)

Considering the TPR obtained for each subject, it is possible to see that all users reached almost 70% accuracy, although each one achieved his/her best mark with different classifiers. Moreover, the standard deviations have reasonable values in all results, since the training data used were not as good in all iterations performed during the cross-validation. Users A, B and D had their best performancewith some kind of k-NN, and also, their deviation is really small: 85.2% ± 3.5% with 20-NN, 81.9% ± 3.6% with 30-NN and 76.9% ± 3.7% with 10-NN, respectively. In the case of User F, the best one was NB with 86.7% ± 5.6% and Users D and E reached 71.0% ± 10.4% and 74.3% ± 5.4% with SMO, respectively. Focusing our attention on the average of all users, it is possible to see that QP-SVM achieved 71.68% ± 6.2% accuracy above the others. This value has also the smallest deviation, so it indicates that the training data affected the results less than others classifiers.

### 3.2. False Positive Rate (FPR)

As can be observed, FPR for all users and classifiers was above 17%, and in some cases, it reached up to 70%. This is one thing to improve in further research. The ultimate goal of this research is to use the output of the classifier to command an external system that moves the arm of the user depending on whether the user wants to move his/her arm or not. Therefore, if the classifier detects pre-movement when the user wants to remain at rest, the engines of the exoskeleton or FES will be activated, and the user will perform an unintentional movement. It would be an inconvenience to the subject, and surely it will be counter-productive in the process of rehabilitation of the upper limb. However, if the classifier detects pre-movement as resting samples, subjects will only need to try again.

### 3.3. GAP

GAP is an index that indicates the difference between TPR and FPR. This parameter should be higher than one. The larger the index, the better the classification. Most users obtained the best value of this parameter with some kind of SVM classifier. All of them reached a value of two for this index, so this indicates that the sum of powers selected as characteristics of the classifier can be at least determinant to correctly classify the data. It is also true that in some users, like C, E and, above all, F, this value should be higher and reach at least three to be considered in future experiments, because the classifier requires the minimum possible FPR without a bad TPR. The average of the GAP index is better in LS-SVM and QP-SVM with equal values (2.8).

## 4. Conclusions

This research has been done in order to select the most convenient classifier to detect the intention of an arm movement between three families of classifiers usually used in BCI research. Our final objective is to help in the rehabilitation of disabled people to perform this movement with an exoskeleton attached to the upper limb. If the system is able to predict each movement with a really low FPR, the classifier output will be used as a command to activate the engines or the FES system of the exoskeleton, moving the user's arm. The relationship between the cognitive process to perform such movements and the real movements could improve the rehabilitation due to neuroplasticity.

In summary, SVM classifiers obtain the best results with TPR and FPR around 70% and 28%. Although these values are good for these initial tests, the FPR is a bit high. In other works related to the detection of the intention to perform a wrist, an ankle and a reaching movement, the true positive rates are on average 52% [17], 82.5% [18] and 80% [19], respectively. The studies were done using ERD or BP phenomena, and the results were shown in different ways. Therefore, the TPR obtained in this study is a good starting point, but the false positive rate in other studies is around 10%. Therefore, greater efforts should be made to minimize this index. It is important that the classifier has less resting samples detected as pre-movement to be efficient in a rehabilitation process. An undesired performed movement might not improve rehabilitation or even worsen it. In this study, LS-SVM is the best option, since it provides the minimum rate of false positives. However, only six users performed the test. Using a wider population would make it possible to achieve more reliable results. At least, SVM classifiers behave better.

In future works, new tests with healthy subjects will be performed with some possible improvements in processing data in order to decrease the FPR. Usually, EEG signals have some artifacts, e.g., produced by eye blinks or eye movements, that should be removed. Moreover, as the users have to perform movements during the experiment, the EEG signals could be affected by muscle artifacts, and they should be widely considered [36]. Furthermore, a process based on independent component analysis (ICA) [37] or principal component analysis (PCA) or even linear discriminant analysis (LDA) [38] should be tested to automatically extract the features of the EEG signals related to the movement onset. Other combinations of frequency sums in the mu and beta bands and more sorts of frequency and spatial filters, such as common average reference (CAR), should be tested. Additionally, a Welch method to obtain the power spectra could improve the results obtained with FFT. Afterwards, real-time tests will be carried out to validate the processing and the classifiers. In this sense, the idea is to process the data every 0.5 s with a one-second sliding window. Any SVM classifier is quick enough to make a decision in this time interval. Furthermore, a new set up with 32 electrodes placed mainly in the parietal and frontal lobes will be used, since 16 electrodes would not be enough to remove the EEG signals' noise properly. Moreover, they might not be in the best position to capture the ERD phenomenon, since it is expected that the origin is the motor cortex. Finally, healthy and disabled people will use the best processing and classifier in order to command an exoskeleton attached to the upper limb in an emulated test of a rehabilitation process.

## Figures and Tables

**Table 1.** Results for all users and classifiers. TPR and FPR are in percentages, and GAP is the division between TPR and FPR. SMO, sequential minimal optimization; QP, quadratic programming; NB, naive Bayes.

**Method**	**User**	**A**	**B**	**C**	**D**	**E**	**F**	**All Users**
**LS**	**TPR**	75.7±4.3	77.4±2.3	67.8±6.9	70.2±12.6	72.2±3.2	65.3±5.4	71.4±5.8
	**FPR**	18.5±10.8	26.8±6.0	29.6±7.0	18.8±6.2	31.5±7.0	42.7±7.1	28.0±7.4
	**GAP**	4.1	2.9	2.3	3.7	2.3	1.5	2.8

**QP**	**TPR**	78.5±2.3	76.4±2.8	69.2±6.6	70.9±11.7	70.8±2.6	64.3±5.1	71.7±5.2
	**FPR**	17.3±11.0	26.5±5.7	31.2±7.8	22.8±2.7	30.4±5.1	40.7±5.0	28.1±6.2
	**GAP**	4.5	2.9	2.2	3.1	2.3	1.6	2.8

**SMO**	**TPR**	70.8±5.0	72.6±6.1	71.0±10.4	69.1±11.9	74.3±5.4	63.1±6.0	70.1±7.5
	**FPR**	18.8±13.3	23.5±5.2	29.1±8.3	26.9±5.1	32.6±5.8	38.4±4.6	28.2±7.0
	**GAP**	3.8	3.1	2.4	2.6	2.3	1.6	2.6

**10-NN**	**TPR**	82.6±6.4	62.2±2.3	57.3±7.4	76.9±3.7	68.0±3.3	61.4±2.0	68.1±4.2
	**FPR**	23.1±12.8	42.3±6.5	52.8±10.3	45.4±3.3	42.1±5.1	36.1±8.1	40.3±7.7
	**GAP**	3.6	1.5	1.1	1.7	1.6	1.7	1.9

**20-NN**	**TPR**	85.2±3.5	74.1±1.3	56.8±7.2	71.5±4.9	71.0±4.7	60.8±2.2	69.9±4.0
	**FPR**	21.7±12.5	42.5±3.7	60.2±9.5	47.0±6.3	43.6±3.2	41.7±6.6	42.8±7.0
	**GAP**	3.9	1.7	0.9	1.5	1.6	1.5	1.9

**30-NN**	**TPR**	84.0±3.1	81.9±3.6	62.0±5.4	76.5±3.0	72.4±3.1	68.1±4.1	74.1±3.7
	**FPR**	21.5±10.6	50.6±5.6	54.3±11.9	47.1±6.7	43.1±5.4	48.6±10.6	44.2±8.5
	**GAP**	3.9	1.6	1.1	1.6	1.7	1.4	1.9

**NB**	**TPR**	73.3±2.7	80.0±6.9	59.2±11.3	65.6±9.4	71.5±6.0	86.7±5.8	72.7±7.0
	**FPR**	19.9±10.3	32.3±3.8	41.6±5.7	48.7±5.3	40.6±10.0	70.6±7.4	42.3±7.1
	**GAP**	3.7	2.5	1.4	1.4	1.8	1.2	2.0

**Table 2.** Significance difference in FPR between classifiers.

	**QP-SVM**	**SMO**	**10-NN**	**20-NN**	**30-NN**	**NB**
**LS-SVM**	0.978	0.966	0.050	0.040	0.023	0.099
**QP-SVM**	X	0.988	0.043	0.035	0.020	0.095
**SMO**	X	X	0.035	0.030	0.016	0.090
**10-NN**	X	X	X	0.709	0.549	0.809
**20-NN**	X	X	X	X	0.844	0.954
**30-NN**	X	X	X	X	X	0.826
